# Severe Acute Respiratory Syndrome: Lessons from Singapore

**DOI:** 10.3201/eid0910.030388

**Published:** 2003-10

**Authors:** Kamaljit Singh, Li-Yang Hsu, Jorge S. Villacian, Abdulrazaq Habib, Dale Fisher, Paul A. Tambyah

**Affiliations:** * National University Hospital, Singapore; † Tan Tock Seng Hospital, Singapore

## Abstract

An outbreak of severe acute respiratory syndrome (SARS) occurred in Singapore in March 2003. To illustrate the problems in diagnosing and containing SARS in the hospital, we describe a case series and highlight changes in triage and infection control practices that resulted. By implementing these changes, we have stopped the nosocomial transmission of the virus.

An outbreak of severe acute respiratory syndrome (SARS) was first recognized in Singapore on March 12, 2003. The index patient was hospitalized at Tan Tock Seng Hospital, which has since become the country’s designated SARS hospital. The patient infected 20 other people (including patients and healthcare workers), who subsequently became the sources for secondary spread of the infection (*1*). As of June 12, 2003, a total of 206 cases and 31 deaths attributed to SARS had been reported in Singapore.

We describe the important lessons learned during the triage and containment of SARS at the National University Hospital, Singapore. Both involved expanding isolation criteria to include all patients with undifferentiated fever (even in the absence of respiratory symptoms or chest x-ray changes), improving contact-tracing methods, enforcing the use of fit-tested personal protective equipment in all patient-care areas, avoiding aerosol-generating procedures, and carefully monitoring all healthcare workers for fever or respiratory symptoms. We also highlight the impact of these measures on preventing the entry and nosocomial spread of infection.

## The Study

From March 13 to May 5, 2003, we identified all epidemiologically linked patients whose disease met the Centers for Disease Control and Prevention’s case definition of SARS issued on April 29, 2003 (*2*). Initial investigations included a complete blood count (with a differential count), serum biochemical measurements (including electrolytes, renal and liver function values, creatine kinase, and lactate dehydrogenase), and a chest x-ray. Since the cause of the virus was not known at the onset of the outbreak, routine microbiologic cultures of sputum, urine, and blood were done to rule out common bacterial causes of pneumonia. In addition, mycoplasma serology and urine *Legionella* antigen testing were carried out. When reverse transcriptase–polymerase chain reaction (RT-PCR) kits for coronavirus detection became available, later patients also provided samples for RT-PCR.

Probable SARS was diagnosed in 14 patients and healthcare workers at National University Hospital. The median age of the patients (five men and nine women) was 58 years (range 21–84). Detailed patient characteristics, including background, medical histories, symptoms, and signs, are shown in Tables 1 and 2.

**Table 1:** Admitting clinical characteristics of patients 1 through 7 with severe acute respiratory syndrome^a^

Characteristic	Patient						
	1	2	3	4	5	6	7
Sex	F	F	M	F	F	M	M
Age	71	43	40	78	53	63	84
Source of virus	Community	Community	HCW	Community	Community	Community	Inpatient
Admission diagnosis	Meningitis	Pneumonia	Suspect SARS	Interstitial lung disease	Pneumonia	Congestive heart failure	Pneumonia SARS contact
Co-illnesses	IHD; DM; hypothyroidism	Hypertension		IHD, ypertension; chronic renal failure; CTD on steroids		IHD Hypertension	IHD Cerebrovascular disease
**Symptoms**							
Cough	-	+	+	-	+	+	-
Dyspnea	-	+	-	+	+	-	+
Rhinorrhea	-	-	-	-	-	+	-
Sore throat	-	+	-	-	-	-	-
Headache	-	+	+	-	-	-	-
Myalgia	-	-	+	-	-	+	-
Fever	+	+	+	-	+	+	+
Others	Confusion	Vomiting, diarrhea				Dizziness	
Temp on admission (°C)	38.7	38.9	36.8	36.0	39.6	35.1	38.0
**Physical signs**							
Crackles	+	+	+	+	+	+	+
Chest x-ray findings	Right basal infiltrates	Bibasilar infiltrates	Right basal infiltrates	Right basal infiltrates	Bilateral infiltrates	Bilateral infiltrates	Right upper lobe infiltrates
RT-PCR	ND	ND	Positive	ND	Positive	Positive	ND
Outcome	Survived	Died	Survived	Died	Died	Died	Died

^a^F, female; M, male; HCW, healthcare worker; SARS, severe acute respiratory syndrome; IHD, ischemic heart disease; DM, diabetes mellitus; CTD, connective tissue disease; RT-PCR, reverse transcriptase polymerase chain reaction; ND, not done.

**Table 2:** Admitting clinical characteristics of patients 8 through 14 with severe acute respiratory syndrome^a^

Characteristic	Patient						
	8	9	10	11	12	13	14
Sex	M	F	M	F	F	F	F
Age	63	74	72	28	24	28	21
Source	Inpatient	Inpatient	Inpatient	HCW	HCW	HCW	HCW
Admission diagnosis	Pneumonia; SARS contact	*Escherichia coli* UTI; SARS contact	Pneumonia. SARS contact	Suspect SARS	Suspect SARS	Suspect SARS	Suspect SARS
Co-illnesses	IHD; DM; hypertension	IHD; DM; hypertension; cerebrovascular disease	Dilated cardiomyopathy; hypertension; chronic renal failure	None	None	None	None
**Symptoms**							
Cough	-	-	-	-	-	-	-
Dyspnea	-	-	-	-	-	-	-
Rhinorrhea	-	-	-	-	-	-	-
Sore throat	-	-	-	-	+	-	-
Headache	-	-	-	+	-	-	+
Myalgia	-	-	-	+	+	+	+
Fever	+	+	+	+	+	+	+
Others							
Temp. on admission (°C)	38.5	38.0	38.5	38.2	38.0	38.5	38.8
**Physical signs**							
Crackles	+	-	+	-	-	-	-
Chest x-ray findings	Right basil infiltrates	Bibasilar infiltrates	Right basal infiltrates	Right basal infiltrates	Right basil infiltrates	Right upper lobe infiltrates	Left upper lobe infiltrates
RT-PCR	ND	Positive	Positive	Positive	Positive	Positive	Positive
Outcome	Survived	Died	Survived	Survived	Survived	Survived	Survived

^a^ M, male; F, female; HCW, healthcare worker; SARS, severe acute respiratory syndrome; UTI, urinary tract infection; IHD, ischemic heart disease; DM, diabetes mellitus; RT-PCR, reverse transcriptase polymerase chain reaction; temp., temperature; ND, not done.

### Case Histories

#### Case 2

A woman 43 years of age was admitted to the hospital on March 23; she had had fever, headache, vomiting, and coughing for 7 days and diarrhea on the first day of her illness, which spontaneously resolved. She reported no SARS contacts. The patient was admitted to an isolation room with the diagnosis of community-acquired pneumonia, but her condition rapidly deteriorated. She was transferred to the intensive care unit (ICU), where she died on March 31. On day 3 of her hospital stay, healthcare workers discovered that she had previously visited a friend with hepatitis at the Tan Tock Seng Hospital. Two unidentified SARS patients had been on that hospital ward.

#### Case 3

Case-patient 3 was an ICU physician who performed a bronchoscopy on case-patient 2 on March 26. This procedure was performed in a negative-pressure room with gloves, gown, and an N95 mask. He became ill with fever, headache, and myalgia on March 29. His initial chest x-ray was clear, and his fever resolved transiently for 30 hours before recurring. Subsequent chest x-rays showed right lower-lobe infiltrates that went on to involve both lung fields. He eventually required intubation and ventilatory support in the ICU. He was successfully extubated and has since been discharged.

#### Case 6

A man 63 years of age with coronary artery disease was admitted to the hospital on April 8 after he reported dizziness and shortness of breath. He had seen his general practitioner 2 days earlier with complaints of rhinorrhea, cough, and myalgia. He had a documented temperature of 37.7°C at the physician’s office. On admission he was afebrile, and his chest x-ray showed cardiomegaly with bilateral lung infiltrates. He was admitted to the general medical ward with probable congestive heart failure. However, a transthoracic echocardiogram showed a normal ejection fraction. Within 12 hours, he became critically ill and was transferred to the ICU, where he was intubated. His repeat chest x-ray showed worsening bilateral infiltrates consistent with acute respiratory distress syndrome. The patient had visited an ill brother at the Singapore General Hospital (hitherto a SARS-free hospital). The brother had previously been in Tan Tock Seng Hospital, on March 9–31, and was subsequently identified as the index case-patient for the outbreak at the Singapore General Hospital (*3*). Case-patient 6 went on to infect 15 other people.

#### Case 11

Case-patient 11 was as the on-call physician who assessed case-patient 6 and transferred him to the ICU. She had worn a gown, gloves, and N95 mask, despite no requirement to do so on the general ward at that time. She had fever, headache, and myalgia 3 days later. Her chest x-ray on admission was clear. Respiratory and chest x-ray changes occurred on day 5 of illness; the patient was discharged after 12 days.

### Course of Illness

Most of the patients had a prodrome of fevers and myalgias with no respiratory or chest x-ray changes until several days later. Their illnesses ran a steady course, lasting a median of 11 days. In case-patient 3, the illness exhibited a biphasic pattern with a brief resolution of fever, followed by the return of high temperature and progression of respiratory and chest x-ray changes. A subset of these patients had a fulminant course with rapidly progressing respiratory failure requiring intubation and mechanical ventilation.

### Hematologic and Biochemical Findings

The hematologic and biochemical findings of the case-patients on admission are summarized in Tables 3 and 4. Leukocyte count was normal in nine case-patients. Mild leukopenia was observed in one case-patient, and leukocytosis was observed in another. Lymphopenia (defined as <1.50 X 10^-9^/L) was observed in all patients. We found mild thrombocytopenia in two case-patients. The lactate dehydrogenase levels were elevated in eight case-patients. The C-reactive protein was elevated in 7 of 11 case-patients, and the procalcitonin was raised in four of five case-patients.

**Table 3:** Laboratory data for patients 1 through 7 admitted with severe acute respiratory syndrome^a^

Patient	1	2	3	4	5	6	7
Hemoglobin (g/dL)	**10.9**	12.5	13.4	14.1	12.0	16.3	12.1
Leukocyte count (X10^-9^/L)	4.52	**19.34**	5.3	**14.65**	5.41	9.29	10.14
Lymphocyte count (X10^-9^/L)	**0.78**	**0.94**	**0.71**	**1.02**	**0.53**	**0.63**	**0.21**
Platelet count (X10^-9^/L)	**117**	149	189	180	176	136	**82**
ALT (U/L)	55	37	23	22	**76**	35	**210**
AST (U/L)	**65**	**108**	39	**102**	**121**	**92**	**58**
CK	290	183	213	86	**1,045**	124	**499**
LDH	**747**	**2,513**	**518** ^b^	**1,032**	**2,248**	**2,015**	**955**
C-reactive protein	**5.2**	**34.0**	**141.4**	**27.4**	ND	ND	**7.4**
Procalcitonin	0.14	**1.45**	ND	**1.27**	ND	**15.21**	ND

^a^ALT, alanine aminotransferase; AST, aspartate aminotransferase; CK, creatine kinase; LDH, lactate dehydrogenase; ND, not done; values in boldface are abnormal. ^b^Normal value for LDH at this laboratory was <500.

**Table 4:** Laboratory data for patients 8 through 14 admitted with severe acute respiratory syndrome

Patient	8	9	10	11	12	13	14
Hemoglobin (g/dL)	15.0	8.4	11.2	12.5	14.3	13.2	13.5
Leukocyte count (X10^-9^/L)	3.73	7.2	4.94	6.3	3.6	6.2	6.76
Lymphocyte count (X10^-9^/L)	0.83	1.34	1.04	0.30	0.84	1.13	0.97
Platelet count (X10^-9^/L)	121	163	167	231	176	184	240
ALT (U/L)	51	31	20	10	15	19	8
AST (U/L)	68	52	19	21	21	21	25
CK	35	192	73	68	85	88	56
LDH	685	1,034	390	280	275	319	696
C-reactive protein	3.1	4.3	0.9	<0.7	<0.7	<0.7	<0.7
Procalcitonin	ND	1.27	ND	ND	ND	ND	ND

^a^ALT, alanine aminotransferase; AST, aspartate aminotransferase; CK, creatine kinase; LDH, lactate dehydrogenase; ND, not done.

### Radiologic Changes

We saw a variety of chest x-ray changes in these patients (Tables 3 and 4). The primary abnormalities were patchy unilateral or bilateral consolidation. Opacities were predominantly in the lower lung zones in most patients. Three patients had upper lobe infiltrates. However, five of the patients were admitted to the hospital with normal chest x-ray results.

## Conclusions

On March 18, 2003, our emergency department began screening all febrile patients with respiratory complaints for possible SARS (*2*). All suspected SARS patients were admitted to a negative-pressure isolation room for monitoring. Probable SARS case-patients were transferred to Tan Tock Seng Hospital for further management to keep the hospital free of the SARS virus.

The varied clinical signs and symptoms of SARS have limited the success of such a triage system (*4*–*8*). We have observed that current diagnostic guidelines may not be sufficiently sensitive for assessing patients before admission to hospital. For example, although a temperature of >38°C is part of the diagnostic criteria (*2*), this symptom was notably absent in two patients. The lack of a fever in case-patient 4 may have been the result of long-term steroid use. Case-patient 6 never had a documented temperature >37.7°C and was hypothermic throughout his hospitalization. In addition, a subset of patients may have fever and myalgias without respiratory complaints or chest x-ray changes until later (cases 3 and 11). Booth et al., in a retrospective analysis of 144 patients with SARS, reported that 11% of patients had no respiratory symptoms and would not meet current criteria for SARS, despite having fever, contact history, and chest x-ray infiltrates (*9*). Rainer et al., in a study of 515 patients attending a SARS screening clinic, found that current World Health Organization guidelines, which emphasize respiratory tract symptoms, had a low sensitivity of 26% (*10*). We expanded our isolation criteria to include all patients with undifferentiated febrile illness and chest x-ray infiltrates with or without fever or respiratory symptoms until an alternative diagnosis is made, or until they defervesce and serial chest x-ray findings are normal.

The nonspecific symptoms of this illness often require establishing a history of contact with a SARS patient as a critical clue to the diagnosis (*2*). However, a contact history may not be forthcoming at the initial interview. In Singapore, SARS is an imported infection in which the epidemiology remains well defined with clear lines of secondary spread in the hospitals and community (*3*). Emphasis is placed on using local epidemiologic clues to elicit a history of visits or previous admissions to Tan Tock Seng Hospital or other healthcare facilities and to inquire whether any family members are ill or hospitalized. Patients without a contact history are still isolated if the clinical suspicion is high. A national computerized database of SARS patients and their contacts has been established to assist with this process. An epidemiology team is also used to perform more exhaustive contact tracing and liaisons with other healthcare facilities. This team also monitors and investigates any clusters of pneumonias within our hospital. Suspected case-patients are then immediately transferred to the isolation area for further investigation.

Our initial infection-control policy first required staff working in the emergency department, ICU, and isolation rooms to wear full personal protection equipment (PPE), which included an N95 mask, disposable gloves, and long-sleeve gowns. Virus transmission is likely due to droplet infection, but fecal-oral transmission has also been reported (*11*). The role of fomites has yet to be defined; preliminary data suggest that the virus can remain viable at room temperature for at least 2 days. Recognizing that patients with unsuspected cases of SARS could slip through our triage system onto the general wards (see case 6), all hospital staff with direct patient contact were also required to wear full PPE beginning April 8. In addition, all patients are now examined with the use of dedicated equipment.

Despite the use of PPE, two of our physicians were infected with the virus. The first physician performed an invasive procedure (bronchoscopy) on case-patient 2. The outlet port of the patient’s ventilator was later discovered to have malfunctioned during the procedure, exposing the physician to a large jet of exhaled air. Reports of protected healthcare workers becoming infected during intubation of SARS patients have also emerged from Canada (*12*). The recommendations on the use of PPE are likely insufficient for procedures that may promote aerosolization of respiratory secretions (*13*). Therefore, these procedures are now performed in a negative-pressure room (with an anteroom) using a positive air–purifying respirator suit.

The second physician (case-patient 11) may have become infected because the N95 mask was poorly fitting. Alternatively, transmission may have occurred through the conjunctival mucosa. All healthcare workers are now required to use eye protection when examining patients. Fit-testing of N95 masks is also mandatory, in addition to training on the correct use and disposal of PPE.

SARS has demonstrated remarkably efficient transmission in the hospital environment (76% of Singapore cases were nosocomially acquired) (*3*). At our hospital, nosocomial transmission occurred in nine persons. Our hospital structure of open wards with large numbers of beds separated by curtains may have been a contributory factor to the spread of the virus. These wards are subdivided into cubicles of four to eight beds and have open windows and ceiling fans with no controlled airflow patterns. Another likely factor was the failure to implement a policy of universal PPE use early in the outbreak. Where such measures were implemented (i.e., isolation rooms), no nosocomial transmission occurred.

In addition, because hospital staff are a recognized source of secondary transmission (*3*,*4*,*6*), all healthcare workers are now required to monitor their temperatures three times a day. Anyone with a respiratory illness or a temperature >37.5ºC is removed from duty, pending further evaluation. This policy has successfully prevented the secondary transmission of SARS from affected healthcare workers.

The lessons gathered from our hospital outbreak have resulted in dramatic changes to our triage and infection-control policies. All patients with undifferentiated febrile illness, respiratory complaints, or chest x-ray infiltrates are isolated, screened for SARS contacts, and nursed with full PPE. Despite continued community transmission of SARS in Singapore (the last community case was identified on May 10, 2003), the measures implemented since April 8, 2003, enabled us to identify and contain eight additional SARS cases and prevent the nosocomial transmission of the virus.
